# Clinical Relevance of *FOXP3*, *PD-L1*, *PD-1*, and *miR-155* Gene Expression and Genetic Variants in HPV-Negative Oral Carcinomas

**DOI:** 10.3390/ijms26157218

**Published:** 2025-07-25

**Authors:** Nemanja Ivkovic, Debora Misic, Ruzica Kozomara, Sasa Jovic, Ahmad Sami, Gordana Velikic, Srboljub Stosic, Gordana Supic

**Affiliations:** 1 Institute for Medical Research, Military Medical Academy, 11040 Belgrade, Serbia; 2 Medical Faculty of Military Medical Academy, University of Defense, 11000 Belgrade, Serbia; 3 Clinic for Maxillofacial Surgery, Military Medical Academy, 11040 Belgrade, Serbia; 4 Cellular and Molecular Radiation Oncology Laboratory, Department of Radiation Oncology, Universitätsmedizin Mannheim, Medical Faculty Mannheim, Heidelberg University, 68167 Mannheim, Germany; 5 DKFZ-Hector Cancer Institute at the University Medical Center Mannheim, 69120 Mannheim, Germany; 6 Hajim School of Engineering, University of Rochester, Rochester, NY 14627, USA

**Keywords:** oral cancer, OSCC, *FOXP3*, *miR-155*, *PD-L1*, *PD-1*, gene expression, genetic variants, survival, recurrence

## Abstract

PD-L1, PD-1, FOXP3, and miR-155 are emerging as key modulators of immune evasion and progression of oral squamous cell carcinoma (OSCC). This study investigated the clinical relevance of their gene expression and variants in HPV-negative OSCC. Bulk-tissue mRNA expression was evaluated in 70 patients, while variants in *PD-1* (rs36084323), *PD-L1* (rs822336, rs4143815, copy number variation), *FOXP3* (rs3761548, rs2232365), and *miR-155* (rs767649) were assessed in 134 patients. Expression data were validated using the TCGA cohort of 222 HPV-negative OSCC cases. Low *FOXP3* expression was significantly associated with tumor stage (MMA: *p* = 0.028, TCGA: *p* = 0.025) and poor overall survival (MMA: *p* = 0.0004, TCGA: *p* = 0.019) in both cohorts. Declining *FOXP3* expression correlated with advancing tumor stages, and low *FOXP3* expression was significantly associated with poor survival in advanced stage III–IV tumors (MMA: *p* = 0.001, TCGA: *p* = 0.015), but not early-stage tumors. High miR-155 expression was associated with recurrence (*p* = 0.002) and poor survival in the MMA (*p* = 0.007), but not TCGA cohort. *MiR-155* rs767649 was associated with alcohol consumption (*p* = 0.018). These findings point to *FOXP3* and *miR-155* as potential prognostic biomarkers for HPV-negative OSCC. Stage-specific *FOXP3* expression suggests a dynamic immunoregulatory role, with implications for optimizing immunotherapy timing. Further studies are warranted to resolve cellular context and stage-adapted immune interventions in HPV-negative OSCC.

## 1. Introduction

Oral squamous cell carcinoma (OSCC) is ranked among the fifteen most common cancers worldwide, characterized by poor overall survival and high recurrence rate, with a mortality rate approaching 50% [1]. Despite progress in diagnosis and treatment, OSCC remains an aggressive cancer that continues to be a significant global health burden, particularly in regions with high prevalence of tobacco and alcohol consumption [2], highlighting the urgent need for novel prognostic and therapeutic biomarkers.

### 1.1. Pathogenesis and HPV Status

The pathogenesis of OSCC is multifactorial, involving the progressive accumulation of genetic and epigenetic modifications, as well as changes in gene expression that do not involve alterations in the DNA sequence. These modifications include DNA methylation, histone modifications, and post-transcriptional modulation of gene expression by non-coding RNAs such as microRNAs (miRNAs, miRs) [3]. In oral cancer, genetic and epigenetic changes are primarily driven by environmental and behavioral risk factors, such as smoking, alcohol, and infections with high-risk human papilloma virus (HPV) [4]. HPV-negative oropharyngeal and oral cancers are considered to be biologically distinct entities that exhibit genomic instability and more aggressive clinical behavior than their HPV-positive counterparts [5]. As a result, HPV status has been incorporated into the American Joint Committee on Cancer (AJCC) staging system for oropharyngeal cancers, and its consideration is becoming increasingly relevant for OSCC stratification [6].

### 1.2. Immune Response and Checkpoint Mechanisms

Emerging evidence suggests that the anti-tumor immune response, guided by both innate and adaptive immunity, plays a critical role in the development, progression, and therapy resistance of OSCC. This response is initiated by the presentation of tumor antigens by dendritic cells—professional antigen-presenting cells (APCs), which activate naive T lymphocytes: CD4^+^ helper T cells (Th1 and Th2) and CD8^+^ cytotoxic T lymphocytes (CTLs). While CTLs directly destroy tumor cells, Th1 cells enhance anti-tumor immune responses by secreting proinflammatory cytokines such as interferon gamma (IFN-γ) [7,8,9,10]. Under physiological conditions, immune checkpoint molecules such as CTLA-4 (Cytotoxic T-Lymphocyte-Associated Protein 4), PD-1 (Programmed Cell Death Protein 1), and its ligands PD-L1 and PD-L2 maintain immune homeostasis by limiting excessive activation of T-cells, thereby preventing excessive immune activation and tissue damage. The PD-1, encoded by the PDCD1 gene, is expressed on activated T-cells, while its ligands, PD-L1 and PD-L2, encoded by CD274 and CD273, are expressed in both immune and tumor cells. These molecules transmit inhibitory signals that suppress T cell proliferation and enhance the activity of regulatory T-cells (Treg), thereby suppressing immune responses [9,10]. However, tumors exploit this mechanism to evade immune surveillance, and PD-L1 and PD-1 molecules have emerged as key regulators of tumor immune evasion [8,11,12]. PD-L1 is commonly overexpressed on tumor cells in multiple cancers, including head and neck squamous cell carcinoma (HNSCC) and OSCC, where it interacts with PD-1 on T-cells to suppress their cytotoxic activity [13,14]. Proinflammatory cytokines and chemokines within the tumor microenvironment induce PD-L1 expression, which plays a key role in Treg-mediated immunosuppression, creating an immunosuppressive niche. Consequently, the immunosuppressive tumor microenvironment promotes tumor progression by shifting the immune response from activation to tolerance and tumor immune evasion [11,12].

### 1.3. Regulatory T Cells and FOXP3 as Prognostic Modulators

Initially, Tregs were identified by their expression of CD25, a high-affinity receptor that binds interleukin-2 (IL-2) and thereby deprives the T-lymphocytes of the stimulating effect of this cytokine [15,16]. However, CD25 is also expressed in activated T cells, and FOXP3, a member of the forkhead box family of transcription factors, has been established as the ultimate marker and master regulator of Tregs development and function [17]. FOXP3+CD4+CD25+ Tregs mediate immune tolerance and inhibit effector responses via multiple mechanisms, and their infiltration in tumors is associated with immune escape and poor clinical outcome [16]. In addition, FOXP3+ Treg infiltration was associated with increased PD-L1 expression in multiple cancer types, suggesting a potential synergistic role in the tumor immune evasion [18,19]. However, the prognostic impact of *FOXP3* is controversial with a number of studies highlighting its association with the poor prognosis of OSCC and HNSCC patients [20,21], while others indicate the association of *FOXP3* with a longer overall survival [22,23,24].

### 1.4. MicroRNA-155: Dual Role in Tumorigenesis and Immune Regulation

Emerging evidence highlights the role of microRNAs in malignant transformation, progression, and invasion. One of the most studied oncogenic miRNAs, miR-155, is commonly overexpressed in several tumor types, including OSCC [25,26]. MiR-155 not only regulates tumor growth and metastasis [27] but also modulates the anti-tumor immune response by influencing the PD-1/PD-L1 axis [28], suggesting its potential clinical utility as a candidate biomarker of disease progression and treatment response.

### 1.5. Immune Gene Polymorphisms in OSCC: Clinical Implications

Several polymorphisms in immune-regulatory genes, including PD-1 rs36084323, PD-L1 rs822336 and rs4143815, PD-L1 copy number variation (CNV), FOXP3 rs3761548 and rs2232365, and miR-155 rs767649, have been implicated in cancer susceptibility and prognosis. However, their role in HPV-negative OSCC has mainly been underexplored so far. The *PD-1* polymorphism rs36084323 (A>G), located in the promoter region, has been associated with prognosis in multiple cancer types, including esophageal cancer [29]. Polymorphisms in the 3′-untranslated region (UTR) of the *PD-L1* gene, such as rs822336 (G>C) and rs4143815 (C>G), have been associated with the expression and prognosis of *PD-L1* in non-small cell lung cancer (NSCLC) [30,31]. Copy number variation (CNV) in the *PD-L1* gene may also affect its expression, tumor immunity, and prognosis, as demonstrated in NSCLC [32]. Among *FOXP3* polymorphisms, rs3761548 (C>A) and rs2232365 (A>G), have been associated with cancer risk and immune escape in oral carcinoma [33]. The *miR-155* rs767649 (T>A) variant, which may impact its expression and function, was previously associated with the prognosis of NSCLC [34], although its relevance in OSCC remains unclear [35].

### 1.6. Immunotherapy Context and Study Rationale

Over the past few years, cancer therapy with immune checkpoint inhibitors (ICIs), particularly those targeting the PD-1/PD-L1 axis has shown encouraging results in multiple cancer types, including HNSCC and OSCC [11,12]. Although high PD-L1 protein expression correlates with response to ICIs, the effects are variable and only a subset of HNSCC patients show a sustained benefit [36]. This emphasizes the need for new prognostic and predictive biomarkers that could identify patients who may benefit from ICIs therapy.

Thus, our study aimed to investigate the association of mRNA expression and variants in candidate genes *PD-1*, *PD-L1*, *FOXP3*, and *miR-155* with clinicopathological features, prognosis, and survival in HPV-negative OSCC patients. Because of their critical role in regulating the immune response and inflammation, these biomarkers may provide valuable insights into the anti-tumor immune response, particularly as the number of studies specifically focused on HPV-negative OSCC is still limited.

## 2. Results

The OSCC Military Medical Academy (MMA) cohort consisted of 134 patients diagnosed with oral carcinoma who underwent surgical treatment between 2017 and 2022 at the Clinic for Maxillofacial Surgery of the Military Medical Academy in Belgrade. The basic demographic and clinical characteristics of our experimental HPV-negative OSCC cohort (MMA cohort) are shown in Table 1 and Table S1. The majority of patients were male (80%, n = 107), with a median age of 58 years. Regarding smoking status, 75% (n = 101) were smokers, while 25% (n = 33) were non-smokers. Additionally, 75% (n = 100) of patients reported alcohol consumption, while 25% (n = 34) reported abstinence. The independent TCGA dataset for the expression analysis of candidate genes included 222 HPV-negative oral cancer samples. The prevalence of smoking was significantly higher in the MMA cohort compared to the TCGA cohort (*p* = 0.00003). In the MMA cohort, 82.9% (58/70) of patients were smokers, whereas 55.4% (123/222) of patients in the TCGA cohort were smokers. Although the prevalence of alcohol consumption was also higher in the MMA cohort (51/70, 72.9%) compared to the TCGA cohort (144/222, 64.9%), this difference did not reach statistical significance (*p* = 0.246).

### 2.1. Association of Gene Polymorphisms with Demographic and Clinicopathological Features

Table 1 shows the distribution of *PD-1* rs36084323, *PD-L1* rs822336, *PD-L1* rs4143815, *PD-L1* gene CNV, *FOXP3* rs3761548, *FOXP3* rs2232365, and *miR-155* rs767649 polymorphisms, in relation to demographic and clinicopathological features in the MMA cohort (n = 134). None of the investigated genetic variants showed a statistically significant association with sex, age, or smoking. The *miR-155* rs767649 variant was significantly associated with alcohol consumption (*p* = 0.018). The rs36084323 *PD-1* variant was marginally associated with alcohol consumption (*p* = 0.055) and this association was significant when variant allele carriers, combined heterozygote (ht) and homozygous (hom) genotypes, were compared to the wild-type genotype (wt vs. ht + hom) (*p* = 0.038).

No statistically significant association was found between any of the examined genetic variants and nodal status, tumor stage, or tumor size. Minor allele carriers of the *PD-L1* rs4143815 polymorphism (wt vs. ht + hom) indicated a tendency toward increased tumor recurrence risk (*p* = 0.053).

### 2.2. Association of Gene Expression Profiles with Clinicopathological Features in MMA and TCGA Cohorts

In patients with available tumor expression data (n = 70) from the MMA cohort, PD-1, PD-L1, FOXP3 and miR-155 gene expression levels were assessed in relation to demographic and clinicopathological features. Relative fold changes in target gene expression were calculated using the comparative 2^−ΔΔCt^ method and normalized to the GAPDH reference gene, which was selected according to the NormFinder and BestKeeper algorithms. The differences across clinical groups were analyzed using non-parametric tests.

The median level of *FOXP3* expression was significantly associated with tumor size and was higher in smaller tumors compared to advanced tumor sizes (T1/2 vs. T3/4, median values of 4.683 and 1.230, respectively, *p* = 0.007), see Table 2. Furthermore, the expression of *FOXP3* was significantly associated with recurrence status, with a lower expression observed in patients with a positive recurrence status compared to patients without a recurrence (median: 2.107 vs. 7.777, respectively, *p* = 0.002). Conversely, *miR-155* expression was significantly higher in recurrent cases (*p* = 0.002, Mann–Whitney U test). A trend toward higher *PD-1* expression was observed among patients younger than 58 years (*p* = 0.072). The median expression levels of *PD-1, PD-L1, FOXP3*, and *miR-155* genes did not show a statistically significant relationship with sex, age, smoking status, or alcohol use.

Table 3 shows the association of PD-1, PD-L1, FOXP3, and miR-155 expression with available clinical and demographic data from the TCGA cohort. The TCGA validation cohort revealed that FOXP3 expression was significantly associated with tumor stage (*p* = 0.025), and tumor size (*p* = 0.048). PD-1 expression was significantly associated with age (*p* = 0.015) and tumor size (*p* = 0.036). Additionally, miR-155 expression was associated with sex (*p* = 0.032) age (*p* = 0.043), and tumor stage (*p* = 0.019), see Table 3.

**Figure 1:**
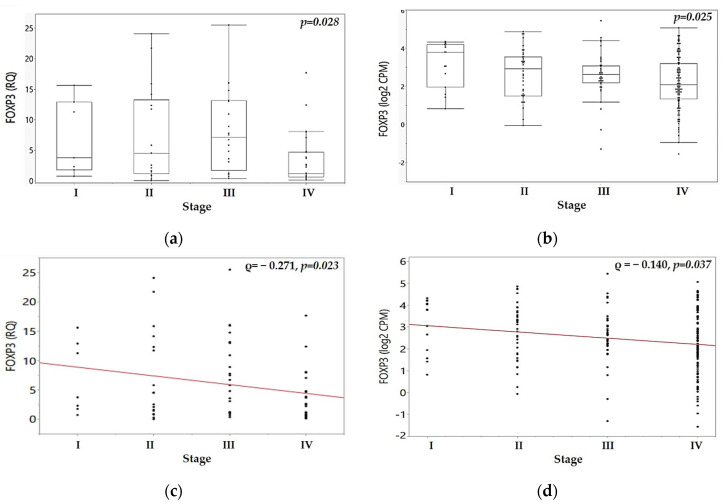
A non-linear relationship of *FOXP3* mRNA expression across tumor stages in OSCC. (**a**) FOXP3 mRNA expression levels across clinical stages I–IV in the MMA cohort; (**b**) *FOXP3* mRNA expression levels across clinical stages I–IV in the TCGA cohort; Spearman correlation, indicated by the red line, between *FOXP3* mRNA expression (shown as individual black dots) and tumor stage in the MMA cohort (**c**) and in the TCGA cohort (**d**). A subset of TCGA patients did not have available clinical stage data (20 out of 222) and therefore were not included in the stage-specific analyses shown in this figure. Mann–Whitney U test or Kruskal–Wallis test were used for statistical analysis, as appropriate.

To evaluate the relationship between *FOXP3* mRNA expression and tumor progression, we analyzed *FOXP3* levels across clinical stages I–IV in both our experimental MMA cohort and the TCGA validation dataset. A significant association was observed between *FOXP3* expression and tumor stage (*p* = 0.028, Kruskal–Wallis test). Median *FOXP3* mRNA expression showed a progressive increase from stage I to stage III tumors in the MMA cohort, see Figure 1a. Expression reached its peak in stage III tumors (median: 7.111) and was significantly higher compared to stage I (median: 3.748, *p* = 0.028, Mann–Whitney U test) or stage II tumors (median: 4.507, *p* = 0.017, Mann–Whitney U test). However, it showed a significant decrease in stage IV tumors when compared to stage III (median: 1.163, *p* = 0.004, Mann–Whitney U test), see Figure 1a. In the TCGA cohort, a similar trend was observed, and stage IV tumors showed a significant decline in *FOXP3* mRNA expression (*p* = 0.025), see Figure 1b. Figure S1 displays mRNA expression of *PD-1*, *PD-L1*, *FOXP3*, and *miR-155* genes across tumor stages I–IV in MMA HPV-negative OSCC cohort, while Figure S2 shows the corresponding mRNA expression profiles in the TCGA HPV-negative OSCC cohort.

Spearman correlation analysis of *FOXP3* mRNA expression across tumor stages revealed a significant negative correlation between tumor stage and *FOXP3* expression in both cohorts, with expression gradually decreasing toward advanced stages. In the MMA cohort, *FOXP3* expression (log_2_ CPM) demonstrated a weak but significant inverse trend (ρ = −0.271, *p* = 0.023; see Figure 1c), while a similar pattern was observed in the TCGA dataset (ρ = −0.140, *p* = 0.037; see Figure 1d).

**Figure 2:**
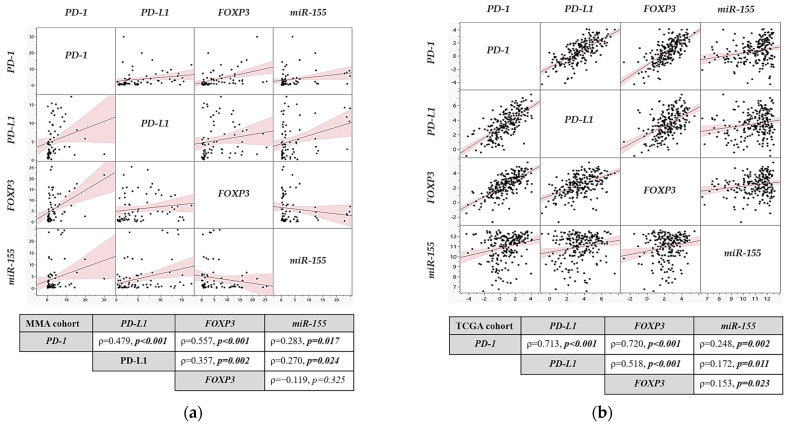
The correlation matrix of *PD-1*, *PD-L1*, *FOXP3* and *miR-155* gene expression in OSCC. (**a**) Spearman’s correlation, indicated by the red line, between the expression of *PD-1*, *PD-L1*, *FOXP3*, and *miR-155* genes (shown as individual black dots) in the MMA OSCC cohort; (**b**) and in the TCGA cohort; ρ (rho)—Spearman correlation coefficient.

To assess the co-regulation of immune-related genes and miRNA, we performed pairwise correlation analyses between *PD-1*, *PD-L1*, *FOXP3*, and *miR-155* in both cohorts. Spearman correlation analysis of gene expression in the MMA cohort showed a statistically significant positive correlation between *FOXP3* and *PD-L1* mRNA expression (ρ = 0.357, *p* = 0.002), *FOXP3* and *PD-1* (ρ = 0.557, *p* = 0.001), and *PD-1* and *PD-L1* (ρ = 0.479, *p* = 0.001), Figure 2a. Additionally, *miR-155* expression correlated positively with *PD-1* (ρ = 0.283, *p* = 0.017) and *PD-L1* (ρ = 0.270, *p* = 0.024), but showed no significant correlation with *FOXP3* expression (ρ = −0.119, *p* = 0.325).

In the TCGA cohort, a stronger positive correlation was observed for all studied genes, including positive correlations between *PD-1* and *PD-L1* (ρ = 0.713, *p* < 0.001), *PD-1* and *FOXP3* (ρ = 0.720, *p* < 0.001), and *PD-L1* and *FOXP3* (ρ = 0.518, *p* < 0.001). The *miR-155* expression was also positively correlated with *PD-1* (ρ = 0.248, *p* = 0.002), *PD-L1* (ρ = 0.172, *p* = 0.011), and *FOXP3* (ρ = 0.153, *p* = 0.023), see Figure 2b.

### 2.3. Kaplan–Meier Survival Analysis

A mean follow-up period in the MMA cohort was 37.43 months, median follow-up was 34.4 months (range: 1–108 months). The mean follow-up period in the TCGA cohort was 30.5 months, with a median of 21.0 months, while the range spanned from 1 to 173 months.

**Figure 3:**
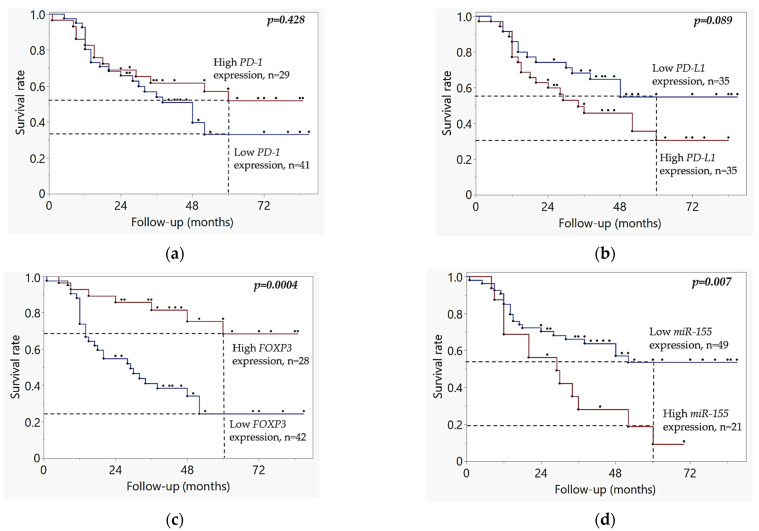
Kaplan–Meier curves based on dichotomized gene expression (high vs. low) in MMA HPV-negative OSCC cohort: (**a**) Overall survival stratified by gene expression status of *PD-1* gene (*p* = 0.428) (**b**) Survival stratified by gene expression status of *PD-L1* gene (*p* = 0.089); (**c**) Survival stratified by gene expression status of *FOXP3* gene (*p* = 0.0004); (**d**) Survival stratified by gene expression status of *miR-155* gene (*p* = 0.007). In the survival curves, the blue line represents high expression, the red line represents low expression, and black dots indicate censored cases. Vertical dashed lines at 60 months (5 years) are included to Kaplan–Meier plots as reference marks to enable a clearer clinical comparison between cohorts. Kaplan–Meier survival curves were analyzed using the log-rank test.

The cutoffs to distinguish samples with low and high mRNA expression levels for *FOXP3*, *PD-L1*, *PD-1*, and *miRNA-155* genes were determined using the ROC_AUC analysis, in both the MMA and TCGA datasets, see Figure S3. The AUC scores and cutoff values in the MMA cohort were as follows: *PD-1* (AUC = 0.54, cutoff = 2.33) *PD-L1* gene (AUC = 0.56, cutoff = 3.65), *FOXP3* (AUC = 0.71, cutoff = 4.81), and *miR-155* (AUC = 0.61, cutoff = 5.47). The AUC scores and cutoff values in the TCGA cohort were as follows: *PD-1* (AUC = 0.52, cutoff = 2.14), *PD-L1* gene (AUC = 0.57, cutoff = 4.06), *FOXP3* (AUC = 0.55, cutoff = 3.05), and *miR-155* (AUC = 0.50, cutoff = 11.78).Kaplan Meier analysis of MMA cohort, Figure 3, demonstrated that patients with high *FOXP3* mRNA expression had significantly better overall survival rates compared to those with low expression (*p* = 0.0004), see Figure 3c. Conversely, high *miR-155* expression was linked to poorer prognosis (*p* = 0.007), see Figure 3d. High *PD-L1* mRNA expression indicated a tendency to be associated with poor survival (*p* = 0.089), see Figure 3b, while *PD-1* mRNA expression levels were not significantly associated with survival (*p* = 0.462), see Figure 3a.

**Figure 4:**
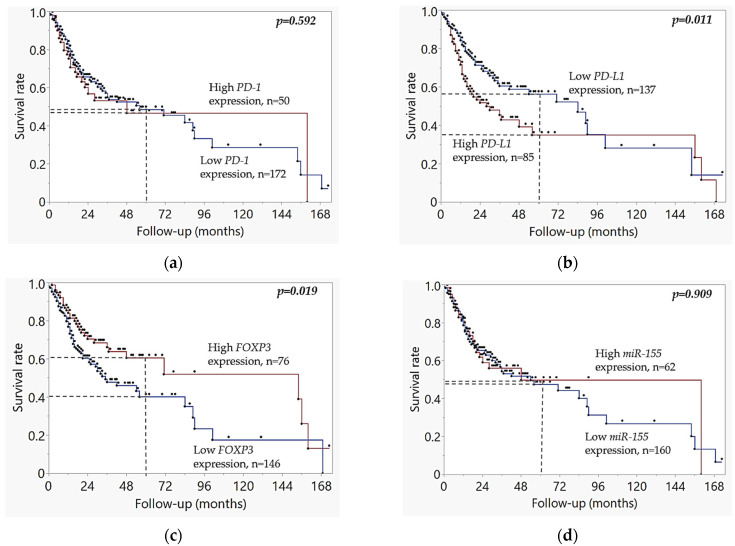
Kaplan–Meier curves based on dichotomized gene expression (high vs. low) in TCGA HPV-negative OSCC cohort: (**a**) Overall survival stratified by gene expression status of *PD-1* gene (*p* = 0.592) (**b**) Survival stratified by gene expression status of *PD-L1* gene (*p* = 0.011); (**c**) Survival stratified by gene expression status of *FOXP3* gene (*p* = 0.019); (**d**) Survival stratified by gene expression status of *miR-155* gene (*p* = 0.909). In the survival curves, the blue line represents high expression, the red line represents low expression, and black dots indicate censored cases. Vertical dashed lines at 60 months (5 years) are included to Kaplan–Meier plots as reference marks to enable a clearer clinical comparison between cohorts. Kaplan–Meier survival curves were analyzed using the log-rank test.

The TCGA dataset analysis confirmed the key findings from the MMA cohort regarding the relationship between *FOXP3* expression and survival, see Figure 4. Kaplan–Meier analysis of the TCGA cohort showed that low *FOXP3* expression was associated with worse overall survival (*p* = 0.019), as seen in Figure 4c, and high *PD-L1* expression correlated with poorer prognosis (*p* = 0.010), as shown in Figure 4b. However, *miR-155* expression was not associated with survival in the TCGA cohort (*p* = 0.909), Figure 4d, in contrast to the findings from our MMA cohort, see Figure 3d. There were no statistically significant differences in survival rates between patients with low and high *PD-1* mRNA expression levels (*p* = 0.592) in the TCGA cohort, as shown in Figure 4a, consistent with the results from the MMA cohort.

When combining *FOXP3* low expression and PD-L1 high expression, *FOXP3*low/*PD-L1*high patients had even more pronounced adverse survival outcome (*p* = 0.000017). Kaplan–Meier analysis of the investigated candidate genetic variants in our OSCC cohort did not reveal any associations with survival.

**Figure 5:**
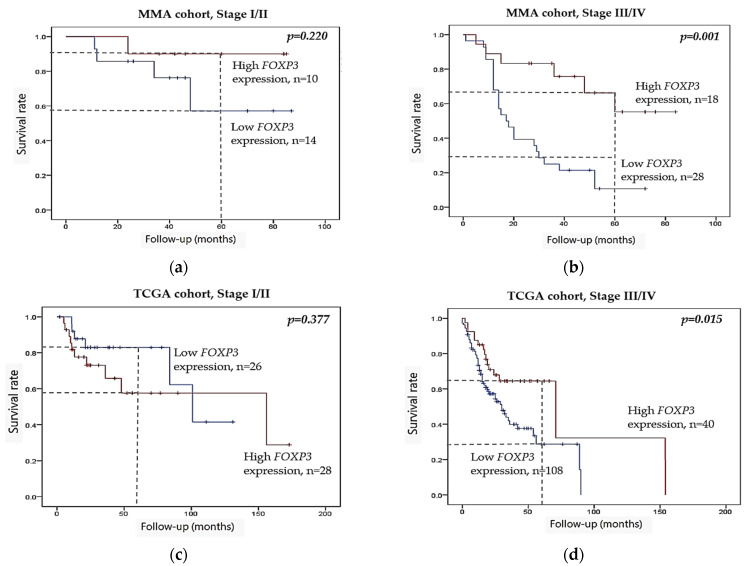
Kaplan–Meier survival analysis of *FOXP3* expression stratified by tumor stage (stage I–II vs. stage III–IV) in the MMA and TCGA cohorts. (**a**) Survival comparison between high and low FOXP3 expression in stage I–II tumors in the MMA cohort. (**b**) Comparison between high and low FOXP3 expression in advanced stage III–IV tumors in the MMA cohort. (**c**,**d**) Corresponding analyses in the TCGA cohort. In the survival curves, the blue line represents high expression, the red line represents low expression, and black dots indicate censored cases. Vertical dashed lines at 60 months (5 years) are included to Kaplan–Meier plots as reference marks to enable a clearer clinical comparison between cohorts. A subset of TCGA patients lacked available clinical stage data (20 out of 222) and therefore were not included in the stage-specific analyses shown in this figure. Kaplan–Meier survival curves were analyzed using the log-rank test.

To further investigate the stage-specific role of *FOXP3*, we conducted stage-stratified Kaplan–Meier survival analyses. While the *FOXP3* expression was not prognostically significant in patients with early-stage tumors, stage I–II (*p* = 0.220), Figure 5a, low *FOXP3* expression was significantly associated with poor overall survival in patients with advanced stage III–IV tumors in the MMA cohort (*p* = 0.001), Figure 5b. A similar pattern was observed in the TCGA cohort, with significant association in advanced stage III–IV tumors (*p* = 0.015), Figure 5d, but not in early stage I–II (*p* = 0.377), Figure 5c.

### 2.4. Cox Regression Analysis

Univariate Cox regression analysis showed that in the OSCC cohort, advanced tumor size (HR = 1.349, *p* = 0.0003), nodal status (HR = 2.168, *p* = 0.007), advanced tumor stage (HR = 2.892, *p* = 0.0000001), and recurrence status (HR = 17.245, *p* = 0.0000001), along with the altered expression of the *FOXP3* gene (HR = 0.252, *p* = 0.001) and the *miR-155* gene (HR = 2.388, *p* = 0.009), were the risk factors for poor survival in these patients. Multivariate regression analysis of the MMA cohort, which included all parameters from the univariate regression analysis with *p* < 0.100, determined that recurrence was an independent predictor of poor survival, HR = 32.126, *p* = 0.000003, see Table 4, while other factors were not statistically significant in the final model.

## 3. Discussion

Oral squamous cell carcinoma (OSCC) is characterized by poor overall survival and high recurrence rate, with a mortality rate approaching 50% [1]. Despite advancements in diagnostics and treatment, significant clinical improvement, particularly for advanced-stage disease, remains unsatisfactory, highlighting the urgent need for novel molecular biomarkers and therapeutic strategies. Presently, cancer immunotherapy that enhances anti-tumor immune response and targets the PD-1/PD-L1 axis has yielded promising results in multiple cancer types, including HNSCC and OSCC [12,37]. Among key regulators of anti-tumor immunity, PD-L1, PD-1, FOXP3, and miR-155 have emerged as key modulators of immune evasion and tumor progression in several malignancies, including OSCC [7,16,38,39]. To contribute to the growing body of research on the regulation of immune checkpoints in cancers, our study focused on assessing the prognostic relevance of the candidate genes *PD-L1*, *PD-1*, *FOXP3*, and *miR-155* in HPV-negative OSCC by integrating gene expression analysis, genetic variations, and patients’ survival.

We observed that low *FOXP3* expression was significantly associated with worse overall survival, tumor recurrence and larger tumor size in our experimental HPV-negative OSCC cohort (MMA), which was validated by the TCGA HPV-negative cohort. Interestingly, declining *FOXP3* mRNA levels correlated with advancing tumor stages, and stage-stratified survival analysis confirmed that low *FOXP3* expression in advanced stages was significantly associated with poor overall survival in both the MMA and TCGA cohorts. In contrast, *FOXP3* expression did not show a significant prognostic effect in early-stage tumors. The observed stage-specific decline in *FOXP3* expression, particularly in stage IV tumors, may indicate immune exhaustion or the loss of immune regulatory mechanisms in late-stage disease. This stage-specific modulation underscores the complexity of FOXP3’s role and suggests that its prognostic relevance may depend not only on expression levels but also on timing within the tumor’s evolutionary trajectory. Additionally, high miR-155 expression was associated with poor survival of OSCC patients, although this trend was not replicated in the TCGA cohort.

Although the ROC/AUC values for FOXP3 and miR-155 expression in our MMA cohort were modest to moderate (FOXP3 AUC = 0.71; miR-155 AUC = 0.61), their association with survival was statistically significant in Kaplan–Meier and univariate Cox analyses. This suggests that while their standalone predictive performance may be limited, their prognostic relevance becomes more apparent when evaluated in the broader clinical and molecular context, particularly in HPV-negative OSCC and advanced stage disease. These findings highlight the importance of interpreting AUC values in conjunction with clinical stratification and survival modeling.

The prognostic value of FOXP3 remains controversial across different cancer types. Our results align with previous studies showing that high *FOXP3* expression is associated with favorable prognosis in OSCC and HPV-negative HNSCC [23,24], as well as in ovarian and colorectal cancer [40,41,42]. These findings may reflect the high number of FOXP3+-expressing tumor-infiltrating lymphocytes (TILs), previously associated with prognosis in HNSCC [22,24,43]. A high infiltration of FOXP3+ TILs emerged as an independent factor associated with better overall survival in HNSCC patients [22]. A comprehensive meta-analysis found that the prognostic impact of FOXP3+ Tregs varies significantly across cancer types. In tumors with strong inflammatory signatures, such as oropharyngeal, hypopharyngeal, esophageal, and colorectal cancers, high *FOXP3* expression is associated with a favorable prognosis [23,24,41]. Furthermore, patients with a favorable prognosis could have infiltration with immunosuppression-incompetent FOXP3+ Tregs, as previously suggested for HNSCC [22].

The rising trend of *FOXP3* mRNA expression from stage I–III observed in both our MMA and TCGA cohorts may reflect an intensified immune response, potentially due to increased infiltration of immune cells within the tumor environment. These findings challenge the conventional role of FOXP3 as solely a marker of immunosuppressive regulatory T cells, which are essential for preserving peripheral tolerance and limiting excessive immune activation but have been associated with tumor-promoting functions [16]. Previous studies have correlated high infiltration of FOXP3+ Tregs with poor prognosis across several tumor types, including gastric and cervical cancer [44,45]. Contrary to our findings, *FOXP3* overexpression has been associated with lymph node metastasis and shorter overall survival in OSCC and tongue carcinoma [20,21]. These conflicting interpretations may result from the fact that our study assessed mRNA expression in HPV-negative OSCC, whereas these studies examined protein FOXP3 expression and did not report stratification by HPV status.

It is critical to emphasize that FOXP3 is expressed in both immune and tumor cells, as demonstrated in multiple cancer types, including tongue carcinoma [21]. This dual expression may partially contribute to the discrepancies observed in its prognostic role. Our study assessed bulk tumor mRNA which captures expression from both tumor and immune cells, whereas IHC methodology can distinguish between FOXP3+ Tregs and FOXP3+ tumor cells. Thus, these discrepancies between studies could be attributed to differences in cellular origin (tumor cells vs. infiltrating Tregs), methodologies (mRNA vs. protein, bulk tumor tissue vs. single-cell/IHC), and post-transcriptional modifications. Due to post-transcriptional regulation via non-coding RNAs and microRNAs, the pattern of FOXP3 mRNA expression might significantly differ from the pattern of protein expression that affects immunological function. Discrepancies between mRNA and protein expression may reflect distinct regulatory dynamics at the transcriptional versus translational levels, especially given that post-transcriptional and post-translational modifications are essential in the regulation of FOXP3 activity [46].

Our findings suggest that FOXP3 may behave as a context-dependent marker, influenced by cellular source, localization, tumor microenvironment, and potentially its interaction with PD-L1. Notably, despite the limited data available on tumor expression in our study, tumors with low levels of *FOXP3* mRNA and high levels of *PD-L1* mRNA were associated with a more pronounced survival differences, suggesting their potential interplay in tumor immune regulation. This is consistent with our findings of a negative correlation between *PD-L1* and *FOXP3* mRNA expression, as well as a positive correlation between *PD-1* and *PD-L1* expression in both experimental and validation cohorts, suggesting a shared immunoregulatory axis of these genes.

Additionally, it is essential to acknowledge the functional plasticity and heterogeneity of Tregs. While traditionally considered immunosuppressive, emerging studies demonstrate that under specific inflammatory conditions, Tregs can acquire pro-inflammatory or even cytotoxic phenotypes, potentially contributing to anti-tumor immunity. This aligns with recent findings that Tregs may exert tumor-suppressive effects in certain cancer contexts, potentially through mechanisms such as suppression of chronic inflammation, stabilization of effector T cell responses, or direct cytotoxicity toward tumor cells [47,48,49]. Such findings challenge the notion of Tregs as a homogeneous and uniformly suppressive population and underscore that FOXP3+ Tregs are functionally diverse population, where their prognostic impact depends on factors such as their subset composition, activation state, tissue type, and the tumor microenvironment context. In OSCC, particularly in the prognostically adverse HPV-negative subset, such context-dependent roles may help explain the favorable prognosis observed in patients with higher *FOXP3* expression. Therefore, a nuanced understanding of Treg function beyond their canonical suppressive role is crucial for accurately interpreting FOXP3-related prognostic data and refining therapeutic strategies.

The observed phenomenon of a non-linear relationship of *FOXP3* mRNA expression across tumor stages in both our experimental and TCGA validation cohorts warrants further investigation. *FOXP3* mRNA expression levels peaking in stage III tumors and decreasing in stage IV tumors in both cohorts may be the result of high number and/or activity of FOXP3+ Tregs and other immune cells in the immune-reactive tumor microenvironment of stage III. The decline in stage IV might suggest a transition from immune activation to immune exhaustion or dysregulated immune microenvironment unable to sustain Treg-mediated homeostasis or anti-inflammatory control. Such findings emphasize the complexity of FOXP3’s role in OSCC and underscore the need for studies involving spatial transcriptomics and single-cell analyses to disentangle the roles of FOXP3+ cell subsets within the tumor microenvironment. Spatial transcriptomics of primary pancreatic cancer revealed cellular heterogeneity and an enrichment of FOXP3-associated Tregs in the tumor front, suggesting that the immunosuppressive microenvironment promotes metastatic dissemination [50].

Additionally, growing evidence indicates that FOXP3 exists in multiple isoforms with distinct functional roles [51]. The FOXP3 Full-Length (FOXP3FL) is primarily expressed in Tregs and is essential for their immune suppression. In contrast, the isoform lacking exon 2 (FOXP3ΔE2) has been predominantly expressed in tumor cells and is linked to decreased immunosuppressive activity and tumor-suppressive roles in epithelial cancers by repressing oncogenes like HER2 [52]. The isoform lacking exons 2 and 7 (FOXP3ΔE2ΔE7) is expressed in some tumor cells and is associated with the loss of immune regulatory functions by interfering with the activity of the full-length isoform [53]. Our study employed the TaqMan gene expression methodology, which captured total *FOXP3* mRNA expression but not isoform-specific levels. Isoform-specific *FOXP3* detection would require targeted RNA sequencing (RNA-seq). Expression levels of different isoforms might significantly vary among TILs, Tregs, and tumor cells, potentially explaining the divergent clinical associations observed among different studies.

In parallel, we observed that high *miR-155* expression was significantly associated with tumor recurrence and worse survival in our experimental cohort, which is consistent with its previously proposed role as an oncogenic regulator in OSCC. miR-155 upregulation in OSCC has been shown to be associated with poor prognosis and recurrence, primarily in HPV-negative settings [25,26]. The miR-155 overexpression may also contribute to epithelial-mesenchymal transition, invasiveness and tumor aggressiveness, particularly in early-stage OSCC, where it may serve as a biomarker of relapse risk [54]. Mechanistically, miR-155 has been shown to influence PD-1 and PD-L1 expression by targeting upstream regulators such as SOCS1, JAK/STAT, and NF-κB signaling pathways, all of which are critical for immune evasion [55,56]. Elevated miR-155 expression was previously associated with increased PD-L1 levels, suggesting that miR-155 may promote an immunosuppressive tumor microenvironment through *PD-L1* regulation [39]. Our observation that miR-155 expression positively correlates with *PD-L1* and *PD-1* mRNA expression in both experimental and TCGA cohort, but not with *FOXP3* expression, suggests that the *PD-L1–miR-155* regulatory loop might be independent of *FOXP3*. Although no significant association between *miR-155* and *FOXP3* expression was found in our study, the relationship is well established. FOXP3 directly induces transcription of miR-155 by binding to the promoter region of its coding gene, BIC, enhancing Treg suppressive functions [57]. MiR-155, in turn, inhibits SOCS1 to promote the stability of FOXP3+ Tregs, enabling prolonged STAT inflammatory signaling, while simultaneously upregulating PD-L1 expression [57]. This FOXP3-miR-155 axis, although not directly correlated in our cohort, may still be active at the cellular level, contributing to the delicate balance between immune tolerance and immunosuppression in the tumor microenvironment. In addition, while the *miR-155* variant rs767649 was not associated with altered gene expression in our study, it was significantly associated with alcohol consumption. Chronic alcohol exposure has been previously associated with miR-155 up-regulation, which in turn increases TNFα and induces inflammation [58].

Notably, while the miR-155 expression had a clear impact in MMA cohort, it showed no prognostic value in TCGA HPV-negative OSCC cohort. Also, high *PD-L1* expression was significantly associated with poor survival in TCGA cohort, but did not reach significance in MMA cohort. These discrepancies may be attributed to differences in sample size between experimental MMA and TCGA validation cohorts, methodological approaches to gene expression analysis (QPCR vs. RNA-seq), and/or cohort-specific etiological factors. Cohort-specific effects could be attributed to observed differences in smoking and alcohol consumption between MMA and TCGA cohorts. Smoking prevalence was significantly higher in the MMA cohort compared to the TCGA cohort, while alcohol consumption was moderately elevated in the MMA, though not statistically significant. The higher prevalence of smoking and alcohol consumption in MMA cohort reflects known epidemiological pattern of high tobacco use and alcohol consumption in the Serbian population in general and among OSCC patients [59]. These population-specific lifestyle factors are known to influence cancer risks, inflammation, tumor behavior, and potentially, immune-related gene expression, contributing to the differential patterns of miR-155 and *PD-L1* expression across cohorts. These population-specific exposures may help explain inter-cohort variability in biomarker expression and survival outcomes.

While statistical significance was not achieved in our experimental OSCC cohort, we found that higher *PD-L1* mRNA expression was linked to a worse prognosis in the TCGA dataset. The expression of PD-L1 protein is commonly assessed by IHC on FFPE samples of cancer tissues in order to identify patients eligible for ICI therapy [12,37]. Conflicting results that challenge the prognostic value of *PD-L1* [38] can be attributed to the heterogeneity of *PD-L1* expression in OSCC tissue samples since PD-L1 is expressed in both tumor and immune cells, but also to difficulties in standardizing PD-L1 IHC evaluation. In addition, comparative research has shown a significant difference between tissue PD-L1 mRNA and protein expression. The identification of *PD-L1* mRNA expression in tumor tissue from patients whose PD-L1 was not detectable by IHC opens the possibility of using *PD-L1* mRNA clinically when PD-L1 IHC protein is negative [60,61]. Furthermore, the detected discrepancies between tissue *PD-L1* mRNA and protein expression can be attributed to post-transcriptional regulation of gene expression by miRNAs.

### 3.1. Clinical Implications

Our findings carry important implications for clinical practice and future therapeutic strategies in HPV-negative OSCC. The substantial prognostic value of *FOXP3* expression, validated across two independent cohorts, positions it as a potentially valuable biomarker for patient surveillance, and treatment planning. In addition, the stage-specific expression dynamics suggest that the timing of immunomodulatory interventions may be critical, with stage III potentially representing an optimal therapeutic window before immune exhaustion sets in during stage IV disease. This new insight could potentially introduce stage-tailored immunotherapy approaches in HPV-negative OSCC.

It should be noted that despite statistically significant associations with overall survival, the moderate AUC values indicate limited discriminatory power when used in isolation. Therefore, *FOXP3* and *miR-155* may serve better as part of composite biomarker panels rather than standalone predictors, especially when combined with clinicopathological parameters or immune gene signatures. The complex, intricate regulatory networks revealed by our correlation analyses between *FOXP3, PD-L1, PD-1*, and *miR-155* emphasize that multi-marker strategies may outperform single-biomarker approaches in predicting response to immunotherapy. The identification of genetic polymorphisms, particularly the *PD-L1* rs4143815 variant, which was marginally associated with increased recurrence risk, suggests that genetic profiling could potentially enhance risk stratification and personalized treatment approaches.

These insights contribute to the growing evidence supporting precision medicine and underscore the importance of integrative biomarker panels in the development of future immune-based therapeutic protocols. Larger, prospective studies incorporating immunophenotyping, spatial transcriptomics, and longitudinal clinical data will be essential to validate these findings and translate them into actionable clinical tools.

### 3.2. Study Limitations

This study has several limitations that need to be addressed. First, the sample size of the primary MMA cohort was relatively small, which may limit statistical power, and the differences in sample size among cohorts may result in inter-cohort discrepancies. Second, there are questions regarding the cohort’s generalizability across various populations because experimental MMA cohort is derived from a single center. Additionally, difference in smoking prevalence between the MMA and TCGA cohorts may confound direct comparisons. Lifestyle exposures may influence tumor immunobiology and survival independently or in combination with the evaluated biomarkers. While this limits direct generalizability, it also underlines the importance of considering regional demographic and behavioral context when interpreting biomarker data and comparing findings across populations. Also, while the TCGA cohort included a broader follow-up range (1–173 months), the MMA cohort, although smaller in size, was prospectively monitored and demonstrated longer mean follow-up compared to the TCGA cohort (37.4 months vs. 30.5 months) and median follow-up durations (34.4 months vs. 21.0 months). These differences in follow-up completeness may contribute to variability in survival associations across cohorts. Moreover, the gene expression analysis was performed on tumor bulk tissue without resolution of cell types and could not definitively attribute the expression of *FOXP3* and *PD-L1* to specific cell types, particularly since these molecules can be expressed in both immune and tumor cells. Also, functional experiments have not been conducted to confirm the mechanistic roles of *FOXP3* or *PD-L1*, nor have they confirmed the immunohistochemistry at the protein level. As mRNA and protein expression may differ due to post-transcriptional regulation, the lack of parallel IHC data limits the conclusions on protein expression and the clinical applicability of immunotherapy. While the modest AUC values indicate limited standalone discriminatory power, this is consistent with the complex, multifactorial nature of cancer prognosis, where single biomarker alone rarely achieve high predictive accuracy. Future studies should also include mechanistic experiments (e.g., in vitro knockdown) to confirm the biological roles of FOXP3, miR-155, and PD-L1 in modulating the immune response to OSCC. Moreover, while QPCR is a robust method for quantifying targeted gene expression, it does not allow for the detection of transcript isoforms or provide genome-wide profiling and broader transcriptomic insights, unlike RNA-seq, which is used in the TCGA cohorts. Inter-cohort differences may also be attributable to different methodological approaches for gene expression analysis (QPCR vs. RNA-seq). Additionally, future integration of proteomic analyses and functional assays may provide more comprehensive insights into the translational relevance of mRNA expression patterns and understanding of the tumor microenvironment.

### 3.3. Conclusions and Future Directions

In conclusion, our results highlight the prognostic relevance of *FOXP3* expression in advanced-stage tumors and point to a complex interplay between *FOXP3*, *PD-L1*, and *miR-155* in the OSCC anti-tumor immune response. Although preliminary, our findings that low *FOXP3* mRNA expression in tumor tissue of HPV-negative OSCC was associated with recurrence risk and poor survival were replicated by the independent TCGA cohort, providing additional support for future precise investigation of OSCC immune modulation and its translational implications.

One of the most intriguing findings of this study is the non-linear trajectory of *FOXP3* expression across tumor stages, validated in both the MMA and TCGA cohorts. Specifically, *FOXP3* expression peaks in stage III OSCC and then sharply decreases in stage IV, suggesting an inflection point in the tumor immune microenvironment. This observation may reflect a transition from active immune surveillance or regulatory balance in intermediate-stage tumors to immune dysfunction or exhaustion in late-stage disease. This biphasic behavior suggests that intermediate-stage tumors represent an optimal therapeutic window for immune modulation and that timing should be considered in future immunotherapy protocols.

The observed non-linear, stage-specific *FOXP3* expression pattern provides a compelling framework for understanding the shifting immunological landscape in HPV-negative OSCC and may have implications for the optimization for the timing of immunomodulatory therapies. It suggests the existence of a temporally defined immunological window, particularly in stage III tumors, during which the tumor microenvironment remains immunologically active and potentially more responsive to immune checkpoint therapy. These observations highlight the importance of optimal treatment timing for immunomodulatory therapies to maximize efficacy while avoiding extensive Treg suppression in stage IV tumors. Our findings support the integration of *FOXP3* expression as a stage-stratified biomarker to guide therapeutic decision-making.

The correlations between the markers under investigation raise the possibility that a more accurate patient stratification for upcoming therapeutic approaches involving immune checkpoint inhibition or miRNA-targeted therapies could be provided by a combined evaluation of *FOXP3*, *PD-L1*, and *miR-155* expression. MiR-155 may have a potential oncogenic role that could promote immune evasion by upregulating the expression of PD-L1. Moreover, the combined evaluation of *FOXP3*, *PD-L1*, and *miR-155* expression patterns may provide a more nuanced, multimodal biomarker framework that may potentially outperform *PD-L1* expression alone in predicting immunotherapy responsiveness. The observed co-expression and correlation patterns support the existence of a miR-155–PD-L1 immunoregulatory axis, suggesting that miRNA-mediated pathways may modulate immune escape and could represent novel therapeutic targets.

Finally, our findings challenge the conventional view of FOXP3+ Tregs as uniformly immunosuppressive cell population, offering evidence for their potential anti-tumor roles in specific tumor contexts. FOXP3’s dual role as both an immunosuppression regulator and a tumor suppressor in different cancers requires further investigation, especially given its stage-specific expression pattern. Future research should also resolve the cellular context via single-cell analyses or immunophenotyping of FOXP3+ populations during the tumor progression, particularly the distinction between FOXP3-expressing Tregs, TILs, and tumor cells, to elucidate this dynamic with greater precision. Ultimately, integrative analysis combining expression, genotype, and spatial immune profiling may unlock refined, stage-adapted immune interventions in HPV-negative OSCC. Taken together, these insights contribute to the refinement of immunotherapeutic paradigms and emphasize the clinical value of integrated immune profiling in OSCC.

## 4. Materials and Methods

### 4.1. MMA Patient Cohort and Ethical Approvals

This study included 134 patients diagnosed with OSCC who had surgical resections between 2017 and 2022 at the Clinic for Maxillofacial Surgery, Military Medical Academy (MMA), Belgrade, Serbia. Ethical approvals were obtained from the Institutional Ethics Committee of MMA (Approval No. 1494-6) and the Ethics Committee of Medical Faculty of Military Medical Academy, Belgrade, Serbia (Approval No. 52/2024). Tumor tissue samples were obtained during surgery, snap-frozen, and stored at −40 °C for further analyses. All patients were treated naive prior to surgery and subsequently received post-operative radiotherapy, and those with high-risk pathological features also received concomitant cisplatin-based chemotherapy. The median age of the patients at diagnosis was 58 years (range: 37–81 years). Demographic and exposure data (e.g., smoking and alcohol use) were collected via patient questionnaires. Basic clinicopathological characteristics of the MMA cohort of OSCC patients are summarized in Supplementary Table S1.

### 4.2. Sample Processing: HPV Status Determination, SNP Genotyping, CNV Analysis, and Gene and miRNA Expression Analysis

DNA and RNA were isolated using TRI Reagent (Ambion, Austin, TX, USA), according to the manufacturer’s instructions. HPV status was determined using the HPV High-Risk Screen Real-TM Quant kit (Sacace Biotechnologies, Como, Italy), and all tumor samples were found to be HPV-negative. Genotyping of candidate single-nucleotide variants (SNVs) was performed using TaqMan SNP Genotyping Assays (Applied Biosystems, Foster City, CA, USA) for *PD-1* (rs36084323), *PD-L1* (rs822336, rs4143815), *FOXP3* (rs3761548, rs2232365), and *miR-155* (rs767649). *PD-L1* copy number variation (CNV) was assessed using a TaqMan copy number assay and RNase *p* reference in duplex format. Quantification was performed using CopyCaller Software v2.1 (Applied Biosystems, Foster City, CA, USA), and CNV ≥ 3 was considered abnormal, as previously described [32]. Table S2. Gene locations and characteristics of genotyped SNVs are summarized in Supplementary Table S2.

Gene expression analysis was performed using reverse transcriptase (RT) and quantitative real-time PCR (QPCR) on a subset of 70 OSCC tumor samples. Total RNA has been reverse transcribed using random primers and the High-Capacity cDNA Reverse Transcription Kit (Applied Biosystems, Foster City, CA, USA). Amplification of *PD-1*, *PD-L1*, and *FOXP3* cDNAs was performed using TaqMan Gene Expression Assays and Gene Expression Master Mix. For miR-155, a specific TaqMan microRNA assay was used, with a stem-loop primer in a reverse transcriptase reaction, and the TaqMan MicroRNA Reverse transcriptase kit (Applied Biosystems, Foster City, CA, USA). Once the RT reactions were complete, the miR-155 RT product was amplified with TaqMan Assay, and No AmpErase UNG Universal PCR Master Mix (Applied Biosystems, Foster City, CA, USA). All gene expression reactions were conducted in triplicate. The selection of the most suitable endogenous reference gene for miRNA normalization was based on findings from our previous study [62], in which we evaluated the expression stability of U6, U44, and U48 in 40 OSCC tumor samples, using the BestKeeper v1.0 (Munich, Germany) and NormFinder v0.953 (Aarhus, Denmark) software [63,64]. We employed the same methodology to select the most suitable endogenous reference gene for mRNA normalization, in which we assessed the expression stability of *GAPDH*, *β-globin*, and 18S rRNA across OSCC tumor samples. While all three genes showed acceptable expression levels and amplification efficiency, *GAPDH* demonstrated a higher stability and lower intergroup and intragroup variations. Relative quantification was performed using the 2^−ΔΔCt^ method and the Pfaffl PCR efficiencies model [65,66], with the sample with the lowest expression level used as a calibrator.

### 4.3. TCGA Validation Cohort

A validation cohort of 222 HPV-negative OSCC samples was obtained from The Cancer Genome Atlas (TCGA), HNSCC project. To ensure demographic and clinical comparability with the OSCC MMA cohort, only samples from treatment-naive patients, white, non-Hispanic/Latino subjects, with confirmed HPV-negative status were included, based on the annotations of the GDAC Broad Institute portal (https://gdac.broadinstitute.org, accessed on 23 March 2024). The TCGA cohort, in line with the MMA cohort, consisted of pre-treatment, primary tumor samples, allowing for valid comparisons of gene expression without the confounding impact of prior therapy. The TCGA cohort did not include a minimum follow-up threshold. After filtering, 222 samples were retained for further analyses of transcriptomic data. The mean follow-up period in the TCGA cohort was 30.5 months, with a median of 21.0 months, and the range spanned from 1 to 173 months.

The raw RNA-seq read counts of the TCGA dataset were filtered for low expression using the edgeR package in R [67]. Data normalization was performed using the voom function in the limma library (version 3.52.2) [68,69]. The expression of mRNA data was extracted for *PD-1*, *PD-L1*, and *FOXP3* genes from the transcription matrix. Expression levels of miRNA-155 were retrieved for the same subset of patients from the processed miRNA-seq data from the TCGA repository.

### 4.4. Statistical Analyses

Statistical data analysis was carried out by SPSS (version 20.0, IBM SPSS Inc., Chicago, IL, USA) and JMP: Statistical Software (version 17, JMP Statistical Discovery LLC, Cary, NC, USA). Associations between variables were tested using the chi-square test or Fisher’s exact test, where appropriate. The association between mRNA expression levels and tumor stage (I–IV) was assessed using the Spearman rank correlation coefficient (ρ), due to the ordinal nature of the tumor stage and the non-parametric expression distribution. The association between mRNA expression levels and tumor stage (I–IV) was assessed using the Spearman rank correlation coefficient, due to the ordinal nature of the tumor phase and the non-parametric expression distribution. A multistep statistical approach was used to assess the association of gene expression with patients’ survival. Logistic regression, consecutive Receiver Operating Characteristic (ROC), and Area Under the ROC Curve (AUC) analysis were utilized to determine the prognostic relevance of candidate gene expression. The optimal cutoff values for dichotomizing gene expression data were defined using the Cutoff Finder platform (https://molpathoheidelberg.shinyapps.io/CutoffFinder_v1/, accessed on 22 July 2025), as previously described [70]. Patients were classified into two groups based on the obtained cutoff values: low and high gene expression. Statistical differences in survival probabilities for low and high gene expression groups were assessed using the Kaplan–Meier survival curves and the log-rank test. The hazard ratio (HR) was calculated by Cox-proportional regression analysis with a 95% confidence interval (CI). All variables statistically significant in the univariate analysis, including those with *p* < 0.100, were jointly studied in the multivariate Cox regression analysis. All the presented *p*-values were two-sided, and the associations were considered statistically significant if *p* < 0.05.

## Figures and Tables

**Table 1:** Association of *PD-1*, *PD-L1*, *FOXP3*, and *miR-155* gene polymorphisms and *PD-L1* gene CNV with demographic and clinicopathological features.

Variables	Total MMA (n = 134)		*PD-1* rs36084323	*PD-L1* rs822336	*PD-L1* rs4143815	*PD-L1* CNV ^1^	*FOXP3* rs3761548	*FOXP3* rs2232365	*miR-155* rs767649
			wt/ht/hom ^2^	wt/ht/hom	wt/ht/hom	No/Yes	wt/ht/hom	wt/ht/hom	wt/ht/hom
Sex	Male	107	95/11/1	42/46/19	43/52/12	78/29	54/3/50	64/4/39	53/54/0
	Female	27	21/6/0	10/10/7	14/10/3	19/8	12/3/12	17/4/6	14/13/0
	*p*/*p* ^3^		0.226/0.202	0.621/1.000	0.519/0.386	0.812	0.173/0.668	0.056/0.828	0.829
Age (median)	<58	63	55/8/0	30/22/11	25/27/11	45/18	31/3/29	39/5/19	30/33/0
	≥58	71	61/9/1	22/34/15	33/34/4	52/19	35/3/33	42/3/26	37/34/0
	*p*/*p* ^3^		0.639/1.000	0.138/0.053	0.095/0.486	0.848	0.989/1.000	0.542/0.860	0.729
Smoking	Never	33	28/5/0	13/14/6	17/11/5	25/8	15/3/15	20/3/10	19/14/0
	Ever	101	88/12/1	39/42/20	40/51/10	72/29	51/3/47	61/5/35	48/53/0
	*p*/*p* ^3^		0.759/0.771	0.979/1.000	0.220/0.314	0.662	0.330/0.690	0.652/1.000	0.316
Alcohol	No	21	15/6/0	6/11/4	9/9/3	17/4	8/1/12	13/0/8	16/5/0
	Yes	113	101/11/1	46/45/22	49/52/12	80/33	58/5/50	68/8/37	51/62/0
	*p*/*p* ^3^		0.055/0.038	0.511/0.339	0.881/1.000	0.432	0.530/0.343	0.439/0.624	0.018
Tumor size	T 1/2	93	80/12/1	41/34/18	37/44/12	64/29	47/4/42	55/6/32	47/46/0
	T 3/4	41	36/5/0	11/22/8	20/18/3	33/8	19/2/20	26/2/13	20/21/0
	*p*/*p* ^3^		0.794/1.000	0.124/0.083	0.495/0.451	0.210	0.903/0.710	0.876/0.704	0.851
Nodal status	N−	50	43/7/0	15/24/11	21/23/6	37/13	26/4/20	28/3/19	23/27/0
	N+	84	73/10/1	37/32/15	36/39/9	60/24	40/2/42	53/5/26	44/40/0
	*p*/*p* ^3^		0.702/1.000	0.272/0.142	0.974/0.858	0.843	0.222/0.721	0.696/0.467	0.296
Tumor stage	I	8	7/1/0	3/3/2	5/3/0	8/0	4/0/4	5/0/3	2/6/0
	II	35	30/5/0	15/14/6	13/17/5	25/10	16/3/16	20/3/12	18/17/0
	III	63	55/7/1	27/23/13	28/28/7	43/20	33/3/27	37/3/23	34/29/0
	IV	28	24/4/0	7/16/5	11/14/3	21/7	13/0/15	19/2/7	13/15/0
	*p*/*p* ^3^		0.967/0.995	0.655/0.402	0.855/0.548	0.296	0.717/0.916	0.894/0.827	0.463
Recurrence	No	58	47/11/0	22/25/11	30/23/5	43/15	28/4/26	34/5/19	25/33/0
	Yes	76	69/6/1	30/31/15	27/39/10	54/32	38/2/36	47/3/26	42/34/0
	*p*/*p* ^3^		0.116/0.127	0.964/1.000	0.165/0.053	0.846	0.496/0.863	0.527/0.725	0.162

^1^ CNV-copy number variation; ^2^ wt–wild type; ht–heterozygote; hom–homozygous genotype; ^3^ *p* values for wt vs. ht + hom. Chi-square test or Fisher’s exact test was used for statistical analysis, as appropriate.

**Table 2:** Association of PD-1, PD-L1, FOXP3, and miR-155 gene expression with demographic and clinicopathological features of the MMA cohort tumor samples.

Variables	Total MMA (n = 70)		*PD-1* Expression Median (25–75%)	*PD-L1* Expression Median (25–75%)	*FOXP3* Expression Median (25–75%)	*miR-155* Expression Median (25–75%)
Sex	Male	58	1.892 (0.718–4.133)	3.905 (1.689–7.933)	3.631 (0.806–10.999)	1.422 (0.568–4.76)
	Female	12	1.531 (0.925–4.335)	2.655 (1.544–10.467)	3.548 (0.982–11.470)	5.465 (0.485–6.689)
	*p*		0.803	0.876	0.827	0.399
Age (median)	<58	35	2.445 (0.979–5.349)	4.897 (1.965–9.334)	4.582 (1.132–11.977)	1.422 (0.502–9.004)
	≥58	35	1.057 (0.599–3.862)	2.655 (1.347–7.201)	2.600 (0.735–10.999)	1.489 (0595–4.959)
	*p*		0.072	0.115	0.279	0.742
Smoking	Never	12	1.531 (0.783–3.599)	6.027 (2.324–11.726)	5.312 (3.046–8.624)	0.871 (0.432–4.556)
	Ever	58	1.891 (0.761–4.790)	3.215 (1.604–7.812)	2.590 (0.806–11.546)	1.538 (0.606–5.71)
	*p*		0.779	0.236	0.454	0.289
Alcohol	No	19	1.845 (0.823–5.603)	4.857 (2.974–7.036)	3.748 (0.903–11.722)	1.417 (0.463–5.468)
	Yes	51	1.588 (0.709–4.106)	2.772 (1.198–9.669)	2.657 (0.822–10.912)	1.641(0.616–16.62)
	*p*		0.602	0.306	0.697	0.627
Tumor size	T 1/2	40	2.032 (0.806–5.332)	3.652 (1.735–8.701)	4.683 (1.659–12.976)	1.344 (0.513–5.239)
	T 3/4	30	1.634 (0.657–3.481)	3.631 (1.074–8.501)	1.230 (0.687–6.125)	1.708 (0.558–6.499)
	*p*		0.419	0.652	0.007	0.307
Nodal status	N−	34	1.369 (0.630–6.182)	3.652 (1.373–6.914)	3.121 (0.882–11.377)	1.454 (0.410–5.467)
	N+	36	2.137 (0.872–3.509)	3.803 (1.899–9.614)	3.769 (0.778–8.041)	1.565 (0.647–5.945)
	*p*		0.742	0.180	0.962	0.417
Tumor stage	I	7	1.032 (0.621–5.603)	1.891 (1.431–4.735)	3.748 (1.779–12.904)	0.595 (0.315–4.959)
	II	17	1.845 (0.715–6.839)	3.645 (1.353–8.739	4.507 (1.183–13.239)	2.336 (0.469–5.561)
	III	20	1.951 (0.889–3.176)	4.199 (2.243–9.614)	7.111 (1.705–13.121)	1.144 (0.398–6.473)
	IV	26	1.763 (0.736–4.456)	4.459 (1.682–8.768)	1.163 (0.637–4.696)	1.641 (0.981–6.654)
	*p*		0.973	0.390	0.028	0.303
Recurrence	No	31	1.845 (0.823–6.351)	3.015 (1.509–7.157)	7.777 (1.463–13.161)	0.733 (0.338–3.561)
	Yes	39	1.588 (0.771–3.466)	4.151 (1.821–10.321)	2.107 (0.733–4.657)	2.593 (0.879–12.51)
	*p*		0.365	0.566	0.002	0.002

Median, and 25th and 75th percentile mRNA expression values. Mann–Whitney U test was used for statistical analysis.

**Table 3:** Association of the *PD-1*, *PD-L1*, *FOXP3*, and *miR-155* gene expression with demographic and clinicopathological features of the TCGA cohort samples selection.

Variables	Total TCGA (n = 222)		*PD-1* Expression Median (25–75%)	*PD-L1* Expression Median (25–75%)	*FOXP3* Expression Median (25–75%)	*miR-155* Expression Median (25–75%)
Sex	Male	143	0.761 (−0.090–2.135)	3.907 (2.729–4.906)	2.733 (1.770–3.676)	11.474 (10.755–11.894)
	Female	79	0.718 (−0.402–1.974)	3.379 (2.286–3.379)	2.275 (1.483–3.285)	11.076 (10.220–11.690)
	*p*		0.390	0.087	0.098	0.032
Age (median)	<58	72	0.505 (−0.527–1.565)	3.623 (2.691–4.857)	2.392 (1.545–3.283)	10.995 (10.279–11.543)
	≥58	150	0.978 (−0.077–2.358)	3.106 (2.268–4.295)	2.467 (1.635–3.498)	11.360 (10.560–11.988)
	*p*		0.015	0.077	0.479	0.043
Smoking	Never	101	0.761 (−0.424–2.099)	3.337 (2.408–4.309)	2.477 (1.591–3.440)	11.127 (10.386–11.911)
	Ever	121	0.735 (−0.100–2.010)	3.465 (2.596–4.817)	2.400 (1.545–3.449)	11.263 (10.414–11.754)
	*p*		0.718	0.381	0.706	0.770
Alcohol	No	75	0.950 (−0.253–2.373)	3.739 (2.646–4.700)	2.433 (1.944–3.478)	11.312 (10.621–11.880)
	Yes	142	0.675 (−0.241–2.011)	3.365 (2.502–4.766)	2.447 (1.470–3.440)	11.153 (10.304–11.889)
	*p*		0.623	0.626	0.499	0.480
Tumor size	T 1/2	93	0.0977 (−0.060–2.434)	3.465 (2.255–4.967)	2.679 (1.626–3.708)	11.318 (10.587–12.070)
	T 3/4	115	0.496 (−0.396–1.724)	3.339 (2.485–4.334)	2.266 (1.520–3.159)	11.065 (10.231–11.690)
	*p*		0.036	0.558	0.048	0.060
Nodal status	N−	87	0.665 (−0.376–2.123)	3.302 (2.301–4.455)	2.435 (1.467–3.497)	11.105 (10.219–11.950)
	N+	102	0.617 (−0.241–1.992)	3.407 (2.458–4.532)	2.396 (1.678–3.409)	11.128 (10.342–11.707)
	*p*		0.716	0.494	0.854	0.837
Tumor stage	I	15	1.609 (0.183–2.613)	4.455 (2.866–5.176)	3.781 (1.947–4.200)	11.498 (10.816–12.070)
	II	39	0.977 (0.051–2.549)	3.462 (2.646–4.964)	2.918 (1.483–3.547)	11.446 (10.636–12.153)
	III	40	1.017 (0.048–2.018)	3.926 (2.697–4.633)	2.629 (2.182–3.069)	11.467 (10.382–11.915)
	IV	108	0.371 (−0.454–1.677)	3.140 (2.344–4.287)	2.078 (1.334–3.199)	10.941 (10.157–11.597)
	*p*		0.038	0.081	0.025	0.019

Median, and 25th and 75th percentile mRNA expression values. Patients for whom clinical staging and/or TNM classification data were not available were not included in this table. Mann–Whitney U test was used for statistical analysis.

**Table 4:** Hazard risk analysis of prognostic factors in relation to overall survival (OS) according to Cox regression analysis in OSCC patients of the MMA cohort sample selection.

Cox Regression Analysis	Variables	OS	
		HR [95% CI]	*p*
Univariate	Sex	0.612 (0.312–1.201)	0.153
	Age (≥median)	0.672 (0.403–1.122)	0.121
	Smoking	1.848 (0.942–3.625)	0.074
	Alcohol	1.478 (0.996–2.194)	0.052
	T 1/2 vs. 3/4	1.349 (1.147–1.586)	0.0003
	Nodal status	2.168 (1.236–3.804)	0.007
	Tumor stage	2.892 (2.008–4.167)	0.0000001
	Recurrence	17.245 (7.812–38.071)	0.0000001
	*PD-1* rs36084323	0.614 (0.291–1.299)	0.202
	*PD-L1* rs822336	1.017 (0.735–1.406)	0.920
	*PD-L1* rs4143815	1.281 (0.909–1.805)	0.158
	*PD-L1* CNV	1.148 (0.679–1.943)	0.606
	*FOXP3* rs3761548	0.939 (0.732–1.204)	0.621
	*FOXP3* rs2232365	0.923 (0.714–1.194)	0.544
	*miR-155* rs767649	0.629 (0.387–1.022)	0.061
	*PD-1* expression	0.691 (0.344–1.388)	0.299
	*PD-L1* expression	1.746 (0.886–3.442)	0.107
	*FOXP3* expression	0.252 (0.109–0.583)	0.001
	*miR-155* expression	2.388 (1.246–4.573)	0.009
Multivariate	Recurrence	32.126 (7.446–138.608)	0.000003

Hazard ratio (HR) for overall survival (OS) in OSCC patients with high versus low mRNA levels of investigated genes. The expression was dichotomized as high and low according to ROC analysis and cutoff values; CI, confidence interval; Cox proportional hazards regression analyses were performed to assess associations with overall survival.

## Data Availability

The data presented in this study are available on request from the corresponding author. The data are not publicly available due to privacy and ethical restrictions.

## Supplementary Materials

The following supporting information can be downloaded at: https://www.mdpi.com/article/10.3390/ijms26157218/s1.

## Abbreviations

The following abbreviations are used in this manuscript:

AJCC	American Joint Committee on Cancer
APCs	Antigen-Presenting Cells
BIC	B-cell Integration Cluster
CI	Confidence Interval
CNV	Copy Number Variation
CTLA-4	Cytotoxic T-Lymphocyte-Associated Protein 4
CTLs	Cytotoxic T Lymphocytes
EMT	Epithelial-Mesenchymal Transition
FFPE	Formalin-Fixed Paraffin-Embedded
FOXP3	Forkhead Box P3
FoxP3FL	Forkhead Box P3 Full-Length Isoform
FOXP3ΔE2	FOXP3 Isoform Lacking Exon 2
FOXP3ΔE2ΔE7	FOXP3 Isoform Lacking Exons 2 and 7
GAPDH	Glyceraldehyde-3-Phosphate Dehydrogenase
HER2	Human Epidermal Growth Factor Receptor 2
HNSCC	Head and Neck Squamous Cell Carcinoma
HPV	Human Papillomavirus
HR	Hazard Ratio
ICIs	Immune Checkpoint Inhibitors
IFN-γ	Interferon Gamma
IHC	Immunohistochemistry
IL-2	Interleukin 2
JAK/STAT	Janus Kinase/Signal Transducer and Activator of Transcription
miRNA (miR)	MicroRNA
MMA	Military Medical Academy
NF-κB	Nuclear Factor Kappa-Light-Chain-Enhancer of Activated B Cells
NSCLC	Non-Small Cell Lung Cancer
OSCC	Oral Squamous Cell Carcinoma
PD-1	Programmed Cell Death Protein 1
PD-L1/2	Programmed Death-Ligand 1/2
QPCR	Quantitative Polymerase Chain Reaction
RNA-seq	RNA Sequencing
ROC/AUC	Receiver Operating Characteristic/Area Under the Curve
RT	Reverse Transcription
SNVs	Single Nucleotide Variants
SOCS1	Suppressor of Cytokine Signaling 1
STAT	Signal Transducer and Activator of Transcription
TCGA	The Cancer Genome Atlas
Th	T helper cell
TILs	Tumor-Infiltrating Lymphocytes
TME	Tumor Microenvironment
TNFα	Tumor Necrosis Factor Alpha
Treg	Regulatory T Cell
UNG	Uracil-N-Glycosylase
UTR	Untranslated Region
