# Supplementary material for: Clinical Relevance of FOXP3, PD-L1, PD-1, and miR-155 Gene Expression and Genetic Variants in HPV-Negative Oral Carcinomas
Source: Int J Mol Sci. 2025 Jul 25;26(15):7218. doi: 10.3390/ijms26157218 (PMC12347458; doi:10.3390/ijms26157218)
Supplement: Supplementary file 1 [file ijms-26-07218-s001.zip › ijms-3702775-supplementary.pdf]

Table S1. Baseline characteristics of MMA cohort.

**Variable**		**MMA cohort (N=134)**	**%**
Sex	Male	107	80%
	Female	27	20%
Age (≥ median)	<58	79	59%
	>58	55	41%
Smoking	Never	33	25%
	Ever	101	75%
Alcohol	No	21	16%
	Yes	123	84%
Histological grade	1	90	67%
	2	29	22%
	3	15	11%
Nuclear grade	1	79	59%
	2	35	26%
	3	20	15%
Tumor size	T1	57	42%
	T2	36	27%
	T3	28	21%
	T4	13	10%
Nodal status	N-	50	37%
	N+	84	63%
Stage	I	8	6%
	II	35	26%
	III	63	47%
	IV	28	21%
Recurrence	No	58	43%
	Yes	76	57%

Table S2. Characteristics of genotyped SNVs.

Protein (Gene)	Gene location	SNV ID	SNV location on GRCh38	Variant type, region	Nucleotide change
PD-1 (PDCD1, CD279)	2q37.3	rs36084323	Chr.2: 241859444	upstream regulatory region	C > T
PD-L1 (CD274)	9p24.1	rs822336	Chr.9: 5448690	intergenic	G > C
		rs4143815	Chr.9: 5468257	3′-UTR	G > C
FOXP3	Xp11.23	rs3761548	Chr.X: 49261784	intron	G > T
		rs2232365	Chr.X: 49259429	intron	C > T
miR-155	21q21.3	rs767649	Chr.21: 25572410	intron	T > A

SNV - single nucleotide variant;

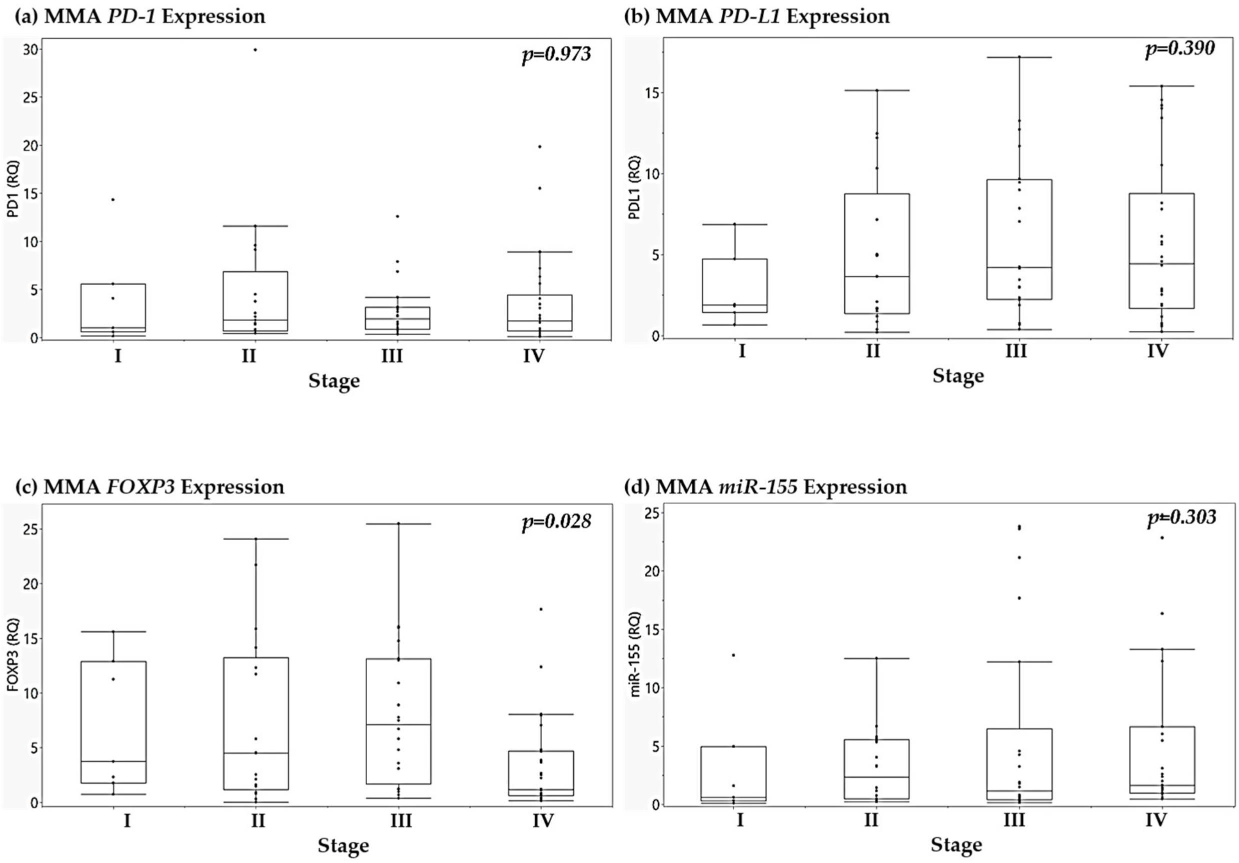

Figure S1. mRNA expression of investigated genes across tumor stages I-IV in MMA HPV-negative OSCC cohort. (a) *PD-1* expression levels across tumor stages I-IV; (b) *PD-L1* expression levels across tumor stages I-IV; (c) *FOXP3* expression levels across tumor stages I-IV; (d) *miR-155* expression levels across tumor stages I-IV; Mann-Whitney U test or Kruskal-Wallis test were used for statistical analysis, as appropriate.

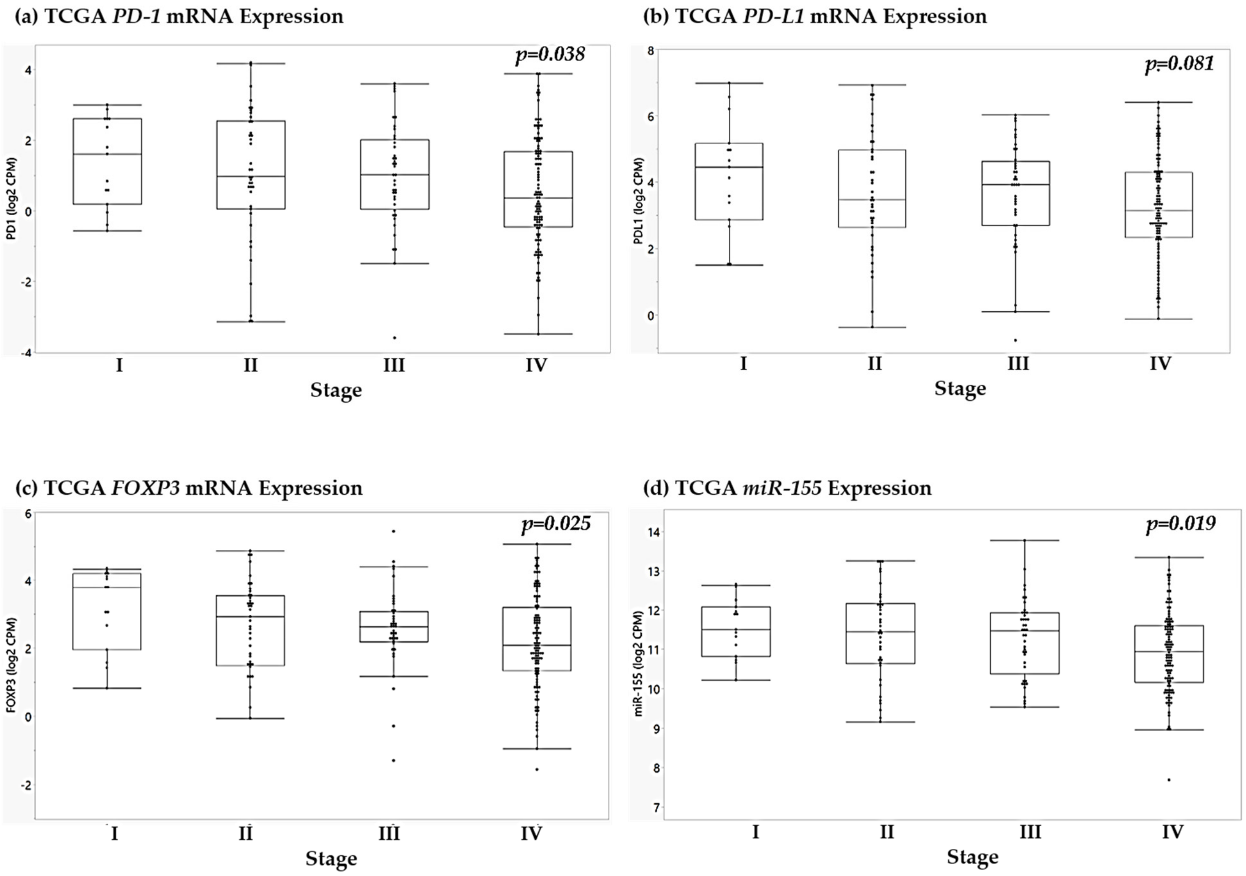

Figure S2. mRNA expression of investigated genes across tumor stages I-IV in TCGA HPV-negative OSCC cohort. (a) *PD-1* expression levels across tumor stages I-IV; (b) *PD-L1* expression levels across tumor stages I-IV; (c) *FOXP3* expression levels across tumor stages I-IV; (d) *miR-155* expression levels across tumor stages I-IV; Mann-Whitney U test or Kruskal-Wallis test were used for statistical analysis, as appropriate.

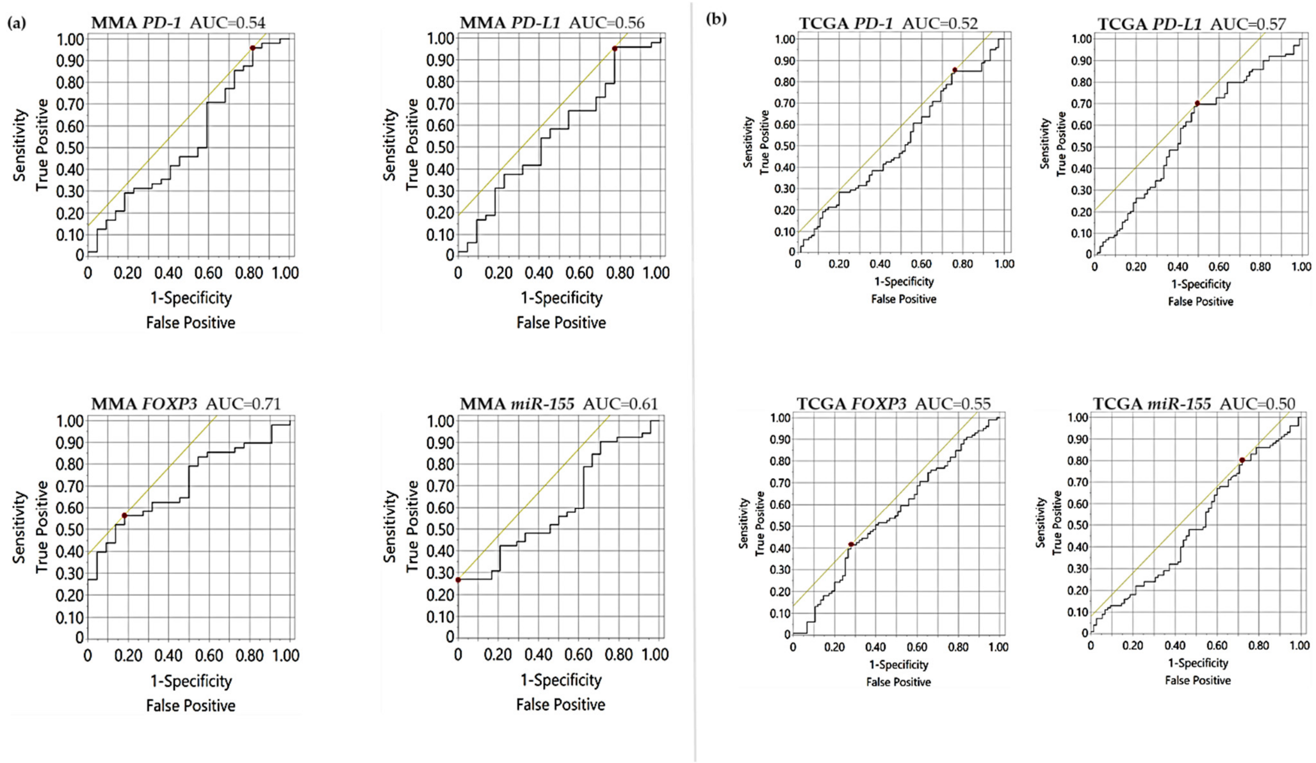

Figure S3. Receiver Operating Characteristic (ROC) curve analysis of *PD-1*, *PD-L1*, *FOXP3* and *miR-155* expression in the (a) MMA and (b) TCGA cohorts. ROC curves were generated to evaluate the discriminatory power of the investigated genes as potential biomarkers for monitoring progression and prognosis in OSCC patients. The area under the curve (AUC) for *FOXP3* in MMA cohort was 0.71, indicating moderate prognostic accuracy, while the AUC for miR-155 in MMA cohort was 0.61, reflecting limited but statistically significant discrimination. The optimal cut-off values for ROC analysis were determined from Youden's index, which maximizes the sum of sensitivity and specificity.
